# Travel-related Schistosomiasis Acquired in Laos

**DOI:** 10.3201/eid1511.090611

**Published:** 2009-11

**Authors:** Eyal Leshem, Eyal Meltzer, Esther Marva, Eli Schwartz

**Affiliations:** Chaim Sheba Medical Center, Tel Hashomer, Israel (E. Leshem, E. Meltzer, E. Schwartz); Tel Aviv University Sackler School of Medicine, Ramat Aviv, Israel (E. Leshem, E. Meltzer, E. Schwartz); Ministry of Health, Jerusalem, Israel (E. Marva)

**Keywords:** Schistosoma mekongi, acute schistosomiasis, Laos, Israel, travelers, parasites, dispatch

## Abstract

Twelve Israeli travelers acquired schistosomiasis in Laos during 2002–2008, and 7 of them had acute schistosomiasis. The patients were probably exposed to *Schistosoma mekongi* in southern Laos, an area known to be endemic for schistosomiasis. Four possibly were infected in northern Laos, where reports of schistosomiasis are rare.

Schistosomiasis is a widely distributed intravascular trematode infection. Estimates indicate that >200 million people are infected with schistosomiasis, mainly in Africa. In Asia, 3 *Schistosoma* species cause human infection: *S. japonicum*, *S. malayensis*, *and S. mekongi*. *S. mekongi* endemicity is thought to be limited to a 200-km area of the Mekong River Basin, stretching from the southern tip of Laos to Cambodia (Figure) (*1*). However, this parasite’s intermediary host, freshwater snails (*Neotricula aperta*), has recently been found to be more widespread and to be advancing northwards (*1*,*2*). Attwood has suggested that *S. mekongi* may extend as far north as Khammouane Province in southern Laos (Figure) (*1*,*3*).

**Figure:**
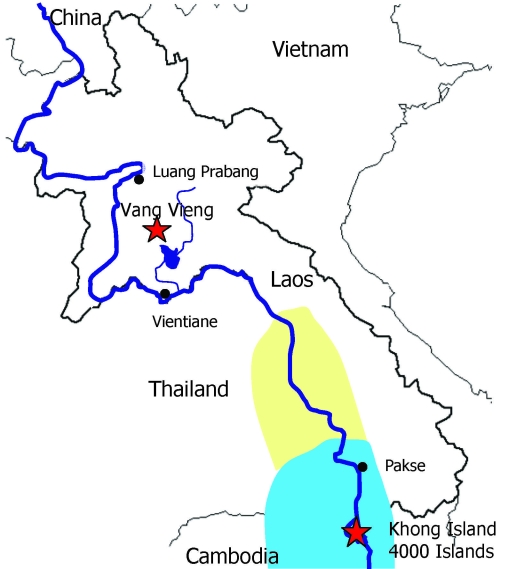
Map of Laos. The area in which *Schistosoma mekongi* is known to be endemic is highlighted in light blue. The area highlighted in light yellow shows both the known area and the area predicted by Attwood’s paleogeographic models (*1*) to be inhabited by *Neotricula aperta* (freshwater snails), the known intermediary host for *S. mekongi*. Two foci of travel-related schistosomiasis are also highlighted with red stars. The dark blue line shows the route of the Mekong River.

Acute schistosomiasis is a transient hypersensitivity reaction associated with tissue migration of *Schistosoma* spp. larvae in nonimmune persons. This syndrome is characterized by fever, cough, fatigue, myalgia, urticaria, and gastrointestinal complaints. Although acute schistosmiasis caused by *S. japonicum* was extensively studied long ago (*4*), we found no reports of acute schistosmiasis caused by *S. mekongi*. Moreover, current literature states that acute schistosmiasis has never been described as a feature of *S. mekongi* infection (*5*).

## The Study

The study was conducted at the Center for Geographic Medicine at Sheba Medical Center and was approved by the institutional review board. Travel-related schistosomiasis was defined as a case of schistosomiasis confirmed by serology or ova detection in a traveler who had been exposed to freshwater in Laos. Travelers were thoroughly questioned regarding freshwater exposures during the index trip and any previous trips to schistosomiasis*-*endemic areas.

Serologic diagnosis conducted at the Israel Ministry of Health (MOH) Central Laboratories in Jerusalem was based on the soluble egg antigen ELISA test (IVD Research Inc., Carlsbad, CA, USA), a nonspecies-specific method. Consequently, most samples (11/12) were sent to the US Centers for Disease Control and Prevention (CDC) for species-specific serologic assays (Falcon Assay Screening Test ELISA [FAST-ELISA], CDC, Atlanta, GA, USA) (*6*). This method is used for *S. japonicum* serodiagnosis; specific serology for *S. mekongi* is unavailable.

Stool specimens were tested for the presence of *Schistosoma* spp. ova (merthiolate–iodine-formaldehyde technique) at the Israel MOH and Clalit Health Services laboratories. Fisher exact test was used for categoric data and the Student *t* test for continuous data. Statistical significance was set at p<0.05.

During 2002–2008, 12 patients (5 male, 7 female [2 children]) had travel-related schistosomiasis acquired in Laos (Table 1). No freshwater exposures in *Schistosoma*-endemic areas in Asia (excluding the index trip to Laos) were reported by patients (Table 1).

**Table 1:** Demographic, epidemiologic, and clinical characteristics of travelers with schistosomiasis acquired in Laos*

Patient no.	Age, y/sex	Clinical features	Countries visited during index trip	Places of water exposure in Laos	Date of exposure	Date of clinic visit	Possible past exposure to schistosomiasis	Serology, genus-specific/ immunoblot	Stool ova
1	27/F	AS	China, Laos, Cambodia, Thailand	4,000 Islands	2003 Apr	2003 Sep	1997, Malawi	Pos/*S. japonicum*	Neg
2	22/F	AS	Thailand, Laos, Cambodia, Vietnam	Vang Vieng, 4,000 Islands	2007 Jun	2007 Aug	No	Pos/*S. japonicum*	Neg
3	23/M	AS	Thailand, Nepal, Laos, Vietnam, Cambodia	Vang Vieng, 4,000 Islands	2006 Apr	2006 Jun	No	Pos/*S. japonicum*	ND
4	38/F	AS	India, Thailand, Laos, Cambodia	Vang Vieng, 4,000 Islands	2008 Apr	2008 May	No	Pos/*S. japonicum*	Neg
5	9/F	AS	India, Thailand, Laos, Cambodia	Vang Vieng, 4,000 Islands	2008 Apr	2008 May	No	Pos/*S. japonicum*	Pos
6	42/M	AS	India, Thailand, Laos, Cambodia	Vang Vieng, 4,000 Islands	2008 Apr	2008 May	No	Pos/*S. japonicum*	ND
7	6/M	AS	India, Thailand, Laos, Cambodia	Vang Vieng, 4,000 Islands	2008 Apr	2008 May	No	Pos/*S. japonicum*	ND
8	24/F	CS	Thailand, Laos, India	Vang Vieng, 4000 Islands	2003 Nov	2006 May	No	Pos/Neg	Neg
9	36/F	CS	India, Thailand, Vietnam, Cambodia, Laos	Vang Vieng	2004 Mar	2005 May	No	Pos/*S. japonicum*	ND
10	26/F	CS	Thailand, Laos, Australia, New Zealand	Vang Vieng	2003 Nov	2004 Nov	No	Pos/*S. haematobium*†	Neg
11	22/M	CS	Thailand, Laos, China, Nepal	Vang Vieng	2007 Feb	2007 Dec	No	Pos/ND	Neg
12	23/M	CDA	Thailand, Laos, Vietnam, Cambodia, China	Vang Vieng	2007 Mar	2007 Aug	No	Pos/*S. japonicum*	ND

*AS, acute schistosomiasis; CS, chronic schistosomiasis; pos, positive; neg, negative; ND, not done; S. *japonicum, Schistosoma japonicum*; *S.* *haematobium*, *Schistosoma hematobium*; CDA, cercarial dermatitis, asymptomatic. †Patient tested positive on *S. haematobium* immunoblot.

Mean patient age was 24 years (range, 6–42 years). Seven patients were exposed to freshwater in both southern and northern Laos (4,000 Islands and the town of Vang Vieng, respectively); 1 patient was exposed only in southern Laos (Figure). Notably, 4 patients reported freshwater exposure exclusively in northern Laos (Vang Vieng). Three of the 4 reported no travel in southern Laos; 1 patient (Table 1, patient 10) visited southern Laos but was not exposed to freshwater. Exposure occurred during the months of February–April for 9 of the 12 patients.

Seven patients had a diagnosis of acute schistosmiasis. Fever (86%), headache (86%), urticarial rash (71%), and cough (71%) were the most prevalent acute schistosmiasis symptoms (Table 2). Four patients reported chronic gastrointestinal symptoms (abdominal pain and discomfort, diarrhea or loose stools). One patient described a pruritic papular rash that appeared hours after exposure and resolved within a few days (suspected cercarial dermatitis). The patient was asymptomatic upon evaluation at our clinic (Table 1, patient 12).

**Table 2:** Clinical characteristics of patients with acute schistosomiasis acquired in Laos compared with those of case-patients from Tanzania*

Clinical characteristic	Infections acquired in Laos, n = 7	Infections among case-patients in Tanzania, n = 19
Fever	6 (86)	13 (68)
Headache	6 (86)†	2 (10)
Urticaria	5 (71)	7 (37)
Cough	5 (71)	15 (78)
Fatigue	4 (57)	11 (58)
Angioedema	3 (42)	2 (10)
Abdominal pain	3 (42)	5 (26)
Diarrhea	2 (28)	7 (37)
Myalgia	2 (28)	7 (37)
Cercarial dermatitis	1 (14)	3 (16)
Time from exposure to seeking medical care, d (±SD)	27 (±4)	38 (±22)
Eosinophil count, cells/mm (±SD)	3,595 (±3,218)	3,535 (±2,394)

*Patients with cases suspected to be caused by *Schistosoma mekongi* infection compared with patients infected with *S. mansoni* and/or *S. haematobium* in Tanzania (*7*). All values are no. (%) except as indicated. †p<0.001.

Diagnosis was made by positive serology in all 12 patients. Eleven serum samples were sent to CDC for speciation; 9 patients had positive immunoblots for *S. japonicum* (Table 1). One patient had a positive immunoblot for *S. haematobium*; this result was judged to be a cross-reaction because the patient had never visited *S. haematobium–*endemic areas.

*S. mekongi/japonicum* ova were detected in stool samples of 1 of 7 patients who submitted such samples for ova detection (Table 1). Issues of technical proficiency and expertise precluded a definite conclusion regarding speciation according to egg size. Laboratory findings in 5 patients with acute schistosmiasis were significant for marked eosinophilia (Table 2).

All infected patients were treated with praziquantel at >12 weeks postexposure to avoid treatment failure (*8*). Of the acute schistosmiasis patients, 3 of the 7 required corticosteroid treatment during the acute illness.

## Conclusions

Acute schistosmiasis, which is considered to be a hypersensitivity reaction that usually develops a few weeks after *Schistosoma* infection, is best studied in nonimmune travelers rather than in continuously exposed local populations. We report 7 cases of acute schistosmiasis presumably caused by *S. mekongi* infection acquired in Laos. Acute schistosmiasis is reportedly not a species-specific phenomenon but may develop after infection with any *Schistosoma* spp (*8*), a view strengthened by this report.

Symptoms of acute schistosmiasis caused by *S. mekongi*, although a small number of cases, appear similar to symptoms of acute schistosmiasis caused by *S. mansoni* or *S. haematobium* (*7*) (Table 2). The only symptom significantly more prevalent in acute schistosmiasis caused by *S. mekongi* was headache.

Most *Schistosoma* infections in travelers are acquired in Africa (*8*,*9*). In our clinic, travel-related schistosomiasis acquired outside Africa was diagnosed only in travelers exposed in Laos (*8*). This exposure is probably due to the popularity of water-related adventure activities among travelers to Laos.

*S. mekongi–*endemic areas in Laos have presumably included only the southern reaches of the Mekong River (Figure) (*1*,*2*,*5*). However, this assumption may reflect a serendipitous effect because schistosomiasis in Laos was first diagnosed in immigrants originating from this region. These early schistosomiasis cases led early epidemiologic surveys to the region of Khong, where most subsequent studies were performed (*5*,*10*). Since these epidemiologic surveys were conducted, *S. mekongi* infections acquired in northern Laos have been described only anecdotally (*11*–*13*).

In this report, we describe 4 patients with schistosomiasis apparently acquired in northern Laos (Figure) after exposure to freshwater exclusively in Vang Vieng; that is, they reported no other freshwater exposure during their visit to Laos. However, because of lack of species-specific serology and the inability to find *Schistosoma* ova in these patients’ stool samples, we can not determine which *Schistosoma* spp. caused their infection.

Most of these patients were infected during February–April, Mekong’s early low-water period, indicating a seasonal infection pattern similar to that of local populations (*5*). The increased risk of schistosomiasis during the late dry season should be conveyed to travelers during pretravel consultations.

Our observation of *Schistosoma* infection in the 4 travelers exposed exclusively in Vang Vieng has several limitations. First, the diagnosis was based on positive serology and not on ova detection. Cross-reactivity of nonhuman *Schistosoma* spp. with *S. japonicum* in serologic studies (e.g., *S. sinensium* or *S. ovuncatum*) could have caused seropositivity in our patients. Second, these 4 travelers (Table 1, patients 9–12) have visited other areas in Asia known to be *Schistosoma* endemic (China) or suspected to be (Vietnam, Nepal). Although travelers were thoroughly questioned regarding possible freshwater exposures, they may not have recalled minor exposures. Finally, we found no published malacologic surveys of the Vang Vieng area, and most experts regard this area as free from *N. aperta*, the intermediate host of *S. mekongi*.

In other world regions (e.g., Lake Malawi in Africa), *Schistosoma*-infected travelers have served as sentinels alerting local authorities to previously unsuspected foci of transmission (*14*). The cases of schistosomiasis in travelers thought to be exposed only in northern Laos, an area where dam building may have changed local conditions, mandates a systematic revaluation of *S. mekongi* distribution in Laos.
